# Recognition and Characterization of Nanoscale Phases: Modulus Mapping of Asphalt Film in Pavement Mixture Cores

**DOI:** 10.3390/polym16172537

**Published:** 2024-09-07

**Authors:** Ming Wang, Yuxuan Wang, Jingxuan Guo, Chengwei Xing, Lingyun Zou, Shuaituan Tian

**Affiliations:** 1 School of Transportation Science and Engineering, Civil Aviation University of China, Tianjin 300300, China; m_wang@cauc.edu.cn (M.W.); wangyuxuan990820@126.com (Y.W.); z970924149@163.com (L.Z.); 2 Key Laboratory for Special Area Highway Engineering of Ministry of Education, Chang’an University, South 2nd Ring Road Middle Section, Xi’an 710064, China; 3 School of Highway, Chang’an University, South 2nd Ring Road Middle Section, Xi’an 710064, China; 4 Beijing Super-Creative Technology Co., Ltd., Beijing 100621, China; tianst@cacc.com.cn

**Keywords:** asphalt film, pavement mixture cores, nanoscale phase modulus, conventional indicators, correlation

## Abstract

The objective of this study is to recognize and characterize the nanoscale phase modulus mapping of the asphalt film in pavement mixture cores using atomic force microscopy quantitative nanomechanical technology. The pavement core samples from the upper and middle layers of four highways and laboratory samples were taken as the research object. The phase modulus–macro property correlation of recovered asphalt was analyzed using mathematical statistics. The results showed that the pavement core samples had more significant multi-phase and diversified phase characteristics compared to lab samples. This indicated that the asphalt in the pavement core had an obvious phase separation phenomenon due to aging. The phase modulus of each sample was distributed across a relatively wide numerical range, and there were also many numerical points with large fluctuations. Especially for the mixture sample containing SBS (Styrene-Butadiene-Styrene)-modified asphalt, the phase modulus distribution mappings presented a multi-peak phenomenon. Hence, considering the distribution characteristics of the data, the box plot method was introduced. Compared with quantified results from laboratory samples, the phase modulus of SBS-modified asphalt increased by 0.96 times, 1.18 times and 1.15 times, and that of base asphalt increased by 0.59 times, 0.56 times, 0.42 times, 1.24 times and 0.39 times, respectively. This indicates that the aging degree of asphalt in the upper layer was generally greater than that of the asphalt in the middle layer and that there was an aging gradient in the direction of pavement depth. All points were within the 95% confidence band in terms of correlation fitting, indicating a better fitting effect between phase modulus and complex shear modulus, as well as between phase modulus and penetration. This research provides innovative ideas for future multi-scale numerical simulation and cross-scale performance model development of asphalt binders.

## 1. Introduction

The aging behavior of asphalt in the mixture determines the service quality and service life of asphalt pavement [1,2]. Xing et al. reported that the aging behavior of modified asphalt was determined by the co-aging rate of the SBS modifier and base asphalt [3]. Similarly, Cui et al. pointed out the composite aging mechanism of SBS-modified asphalt [4]. Sun et al. found that the aging mechanism and effective recycling ratio of SBS-modified asphalt [5]. He et al. systematically analyzed the aging behavior of different types of asphalt binders [6]. Wang put forward an innovative idea of directly judging the aging degree of asphalt while in the mixture [7]. Liu et al. investigated the aging gradient within the bitumen film around the air-binder interface [8]. Guo et al. studied concentration distribution of asphalt binder on aggregate surface using molecular dynamics simulation [9]. Thus, understanding the interfacial behavior between asphalt binder and mineral aggregate is of great importance [10,11]. Tao emphasized the effect of aggregate on the viscoelastic properties of mixtures at different temperatures [12]. With the aging process, mineral factors must have a strong influence on its aging behavior. Therefore, people have been trying to use advanced technical means to directly identify the performance of asphalt film in the mixture, in order to provide technical support for the actual evolution of asphalt properties in the road surface [13].

However, it is not easy to identify the properties of asphalt film directly in the mixture. In recent years many efforts have been made, and some conclusions have been drawn. As reported, it is suitable to test the properties of aggregates in the mixture by using nano-indentation technology [14]. However, Yao et al. studied the aging gradient of mastic in asphalt mixtures based on nanoindentation [15]. Zhu et al. found the interfacial transition zone in asphalt concrete based on nanoscale metrology techniques [16]. Katsuki also investigated the nanoindentation approach characterizing the strain rate sensitivity of the compressive response of asphalt concrete [17]. The results showed that the nanoindentation modulus of asphalt mortar was in better agreement with the aging hardening property of asphalt material, and the reliability and regularity of the test results were not affected by careful and standardized polishing treatment. However, with the deepening of the research, it was found that nanoindentation technology was more suitable for testing the mechanical properties of aggregate components in the mixture due to the limitations of resolution and indenter properties. When nanoindentation technology is used to test soft materials such as asphalt binders, the applied payload may not be able to collect an effective load-displacement curve [14]. Therefore, the application of this technique in the in situ characterization of asphalt film components in the mixture remains to be discussed.

Compared with nanoindentation technology, atomic force microscopy (AFM) is more widely used in the field of asphalt material research. Many researchers have used AFM technology to deeply study the aging response of single asphalt materials. Based on the PeakForce Tapping mode, the German company Bruker developed atomic force microscopy quantitative nanomechanical technology (PF-QNM) [18,19], which opened a new era of fine characterization of phase mechanical properties of asphalt materials in 2012. Das et al. used the PF-QNM mode of atomic force microscopy to test and characterize the nanoscale elastic modulus and adhesion properties of asphalt, and the results showed that aging increased the elastic modulus and decreased adhesion [20]. Poulikakos used AFM technology to observe the asphalt in the aging mixture in the laboratory and found that there was no bee phase (phases of the honeycomb morphology) in the aging asphalt in the mixture specimen [21]. Shen Ju-nan et al. conducted aging tests of different types of mixtures with an all-climate accelerated curing tank and used AFM technology to study the nanomechanical properties of aging asphalt after extraction and recovery [22]. It was found that the nonlinear equation can accurately characterize the aging law of asphalt mixtures from the microscopic point of view. Fischer’s study considered for the first time the mechanical properties of asphalt-phase states under different loading frequencies and further confirmed that there were different phase states at the nanoscale level, and each phase state had different mechanical properties [23]. In addition, Nahar and Schmets studied the microstructure characteristics of asphalt using the PF-QNM mode of atomic force microscopy. The software package Gwyddion (version number 1.1) was selected to analyze the data [24]. It can be seen that researchers have conducted a lot of research in the field of asphalt aging degree identification using the AFM-QNM technology. However, these studies focused on the quantitative characterization of single asphalt-phase morphology and mechanical properties. The phase properties of the asphalt film in the mixtures received less attention.

The evolution behavior of asphalt properties in mixtures is strongly influenced by mineral factors. If the asphalt film properties can be directly identified in the mixture sample, it will provide a strong boost to the study of the aging mechanism of asphalt in service pavement. In order to propose a method to directly characterize the properties of asphalt film in the mixture (i.e., in situ), the mixture core samples of four highways, including the upper layer and the middle layer, were taken as the research object. Using atomic force microscopy and rheological test technology, the in situ recognition and characterization methods of the phase modulus mappings of asphalt film in the mixture were investigated. The correlations between the nanoscale phase modulus and the macroscale properties of the asphalt were analyzed using mathematical statistics. This research provides innovative ideas for the future research of multi-scale numerical simulation or the establishment of cross-scale performance models of asphalt binders.

## 2. Materials and Research Methods

### 2.1. Materials

**Figure 1:**
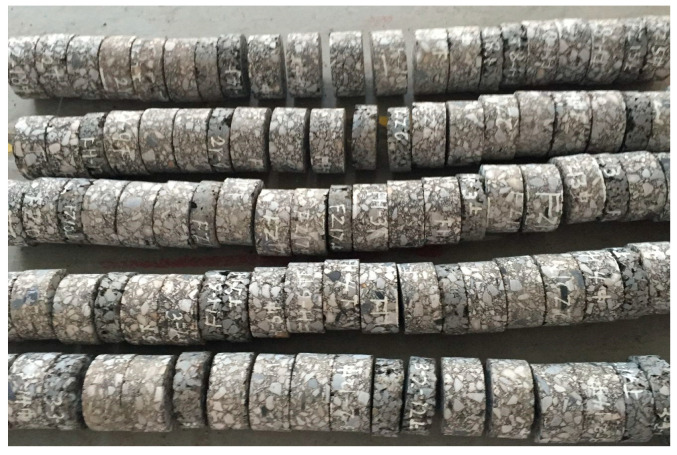
Specimens of pavement core.

The pavement core samples of the upper and middle surfaces (as shown in Figure 1) of four highways (named A, B, C, and D) were taken as the main research objects. According to the pavement information survey, the service life of the four highways was 4~7 years. The pavement structure information and the identification number of the coring sample are shown in Table 1. In order to facilitate comparison, the standard Marshall mixture samples (named M) were molded in the laboratory using the same asphalt type and grading type.

### 2.2. Test Methods

#### 2.2.1. AFM-QNM Technology

**Figure 2:**
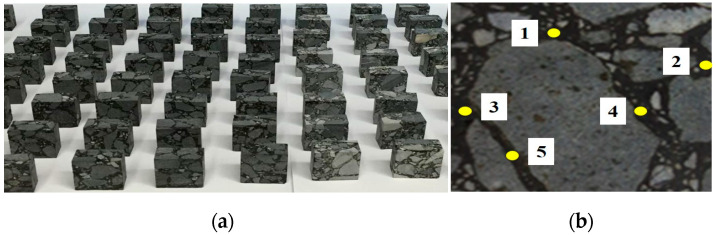
AFM samples and their observation areas. (**a**) Mixtures sample for AFM measurement and (**b**) examples of observation areas from AFM samples.

A dimension icon atomic force microscope, made by the company Bruker in Germany, was used to test the phase modulus of asphalt film in the mixture samples with the QNM mode. As reported in the literature [25], the contact between the probe and the sample in QNM technology is similar to that between a rigid ball and an elastomer. The Derjaguin–Muller–Toporov (DMT) type in contact mechanics is recommended to fit the elastic modulus of the sample (Equation (1)). Considering the viscoelastic properties of asphalt binders, the probe selected in this study was RTESPA, whose elastic constant is 5 N/m. The correction method was the Sader test. The test frequency of this study was 75 HZ, and the scanning range was 5 × 5 μm. For each sample, five scanning areas were selected. The AFM test sample and the observation areas are presented in Figure 2. Figure 2a shows the mix specimen for the AFM test, which was prepared by cryogenic freezing, and the five numbers labelled are its five test zones. In addition, in order to ensure the accuracy of the test results and avoid the difference in the asphalt film properties caused by the asphalt–mineral interaction, the selected scanning areas were far away from the asphalt–aggregate boundary. In addition, professional nanoscope analysis software (v1.8), included with the AFM, was used to analyze the AFM mappings.

(1) $$E={\left(\frac{1-V_{s}^{2}}{E_{s}}+\frac{1-V_{t}^{2}}{E_{t}}\right)}^{-1}$$

where E is the DMT modulus, also known as the discounted modulus, $V_{s}$ and $V_{t}$ are the Poisson’s ratio of test samples and probes, respectively; $E_{s}$ is the calibration modulus of probes, and $E_{t}$ is the modulus of test samples and probes.

The AFM test samples of mixtures, which were prepared by the cryogenic freezing cutting method, are shown in Figure 2a [26]. For each sample, five test areas are measured to ensure the accuracy of the test, as shown in Figure 2b. It should be noted that the test area should avoid the adjacent boundary position of the aggregate because the hard aggregate will destroy the probe tip and affect the accuracy of the test.

#### 2.2.2. Technology for Extracting and Recovering Asphalt from Cored Samples

**Figure 3:**
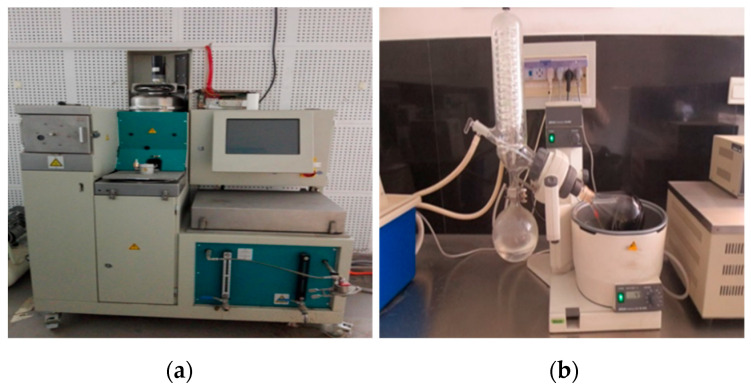
Experimental equipment for extracting and recovering asphalt from cored asphalt mixtures. (**a**) Asphalt mixture analyzer and (**b**) rotary evaporators.

The process of extracting and recovering asphalt from cored mixture samples was as follows: Firstly, the mixture of trichloroethylene and asphalt was obtained by using the asphalt mixture analyzer (shown in Figure 3a), and the other components of the asphalt mixture (aggregate, mineral powder, etc.) were separated. Secondly, the mixed solution was immediately separated using a rotary evaporator (shown in Figure 3b) to obtain the asphalt. It should be noted that the temperature should not be too high in the process of rotary evaporation to avoid secondary aging of asphalt.

#### 2.2.3. Temperature Sweep Test and Penetration Test

Based on AASHTO T315-12 [27], a temperature sweep test was conducted, with a fixed frequency of 10 rad/s, at 60 °C, using a dynamic shear rheometer. For the extracted asphalt, 25 mm diameter and 1mm thickness specimens were prepared. To ensure the reliability of the experimental data, three parallel samples were prepared for the rheological tests. The complex shear modulus G* and phase angle δ were selected to evaluate the viscoelastic properties. In addition, a penetration test was also performed at 25 °C according to the test’s technological specification [28].

## 3. Mapping Recognition and Characterization of Asphalt Phase Modulus

### 3.1. Phase Modulus Mappings Recognition

**Figure 4:**
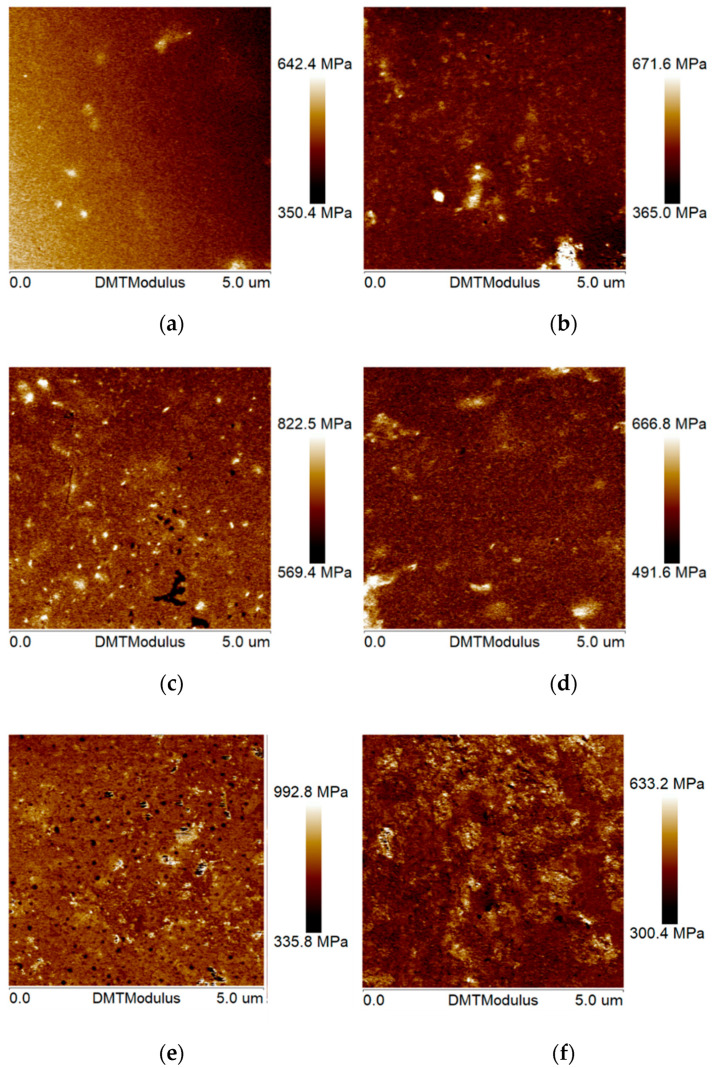

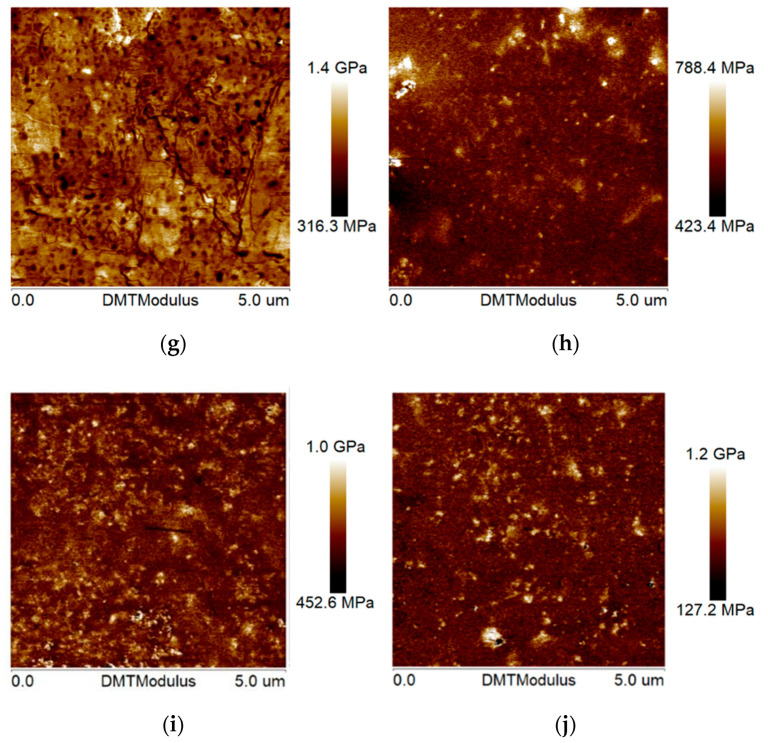
Phase modulus mappings of asphalt in mixtures. (**a**) M-1; (**b**) M-2; (**c**) A-1; (**d**) A-2; (**e**) B-1; (**f**) B-2; (**g**) C-1; (**h**) C-2; (**i**) D-1; (**j**) D-2.

As shown in Figure 4, the DMT phase modulus (also known as Young’s modulus) mappings of the asphalt films in mixtures were obtained using AFM-QNM technology. According to the test principle, the lighter the color, the larger the value of the modulus in the mappings.

It can be clearly seen that, compared with the laboratory samples (namely, M), the characteristics of multi-phase and diversified phase states of the pavement coring samples (namely, A, B, C, and D) were more significant. This phenomenon indicated that the asphalt in the pavement core samples had an obvious phase separation phenomenon due to aging. Specifically, there were two types of phases in the phase modulus mappings of base asphalt. However, three or more phase types were found in the phase modulus mappings of SBS-modified asphalt. Further, it was noted that a new phase similar to black spots was presented in the phase modulus mappings of SBS-modified asphalt, but this phenomenon was not obvious for the base asphalt. This can be explained by the fact that the aging of SBS-modified asphalt is a synergistic occurrence of asphalt-phase aging and SBS-modifier degradation. As a result of different pavement environments, the aging rates of asphalt-phase aging and SBS-modifier degradation may be inconsistent. Some new phase types may also be produced. Similarly, the literature also pointed out that phase separation would occur after the aging of asphalt materials, which was also the key reason for the change in rheological properties and mechanical behaviors of asphalt materials [7].

**Figure 5:**
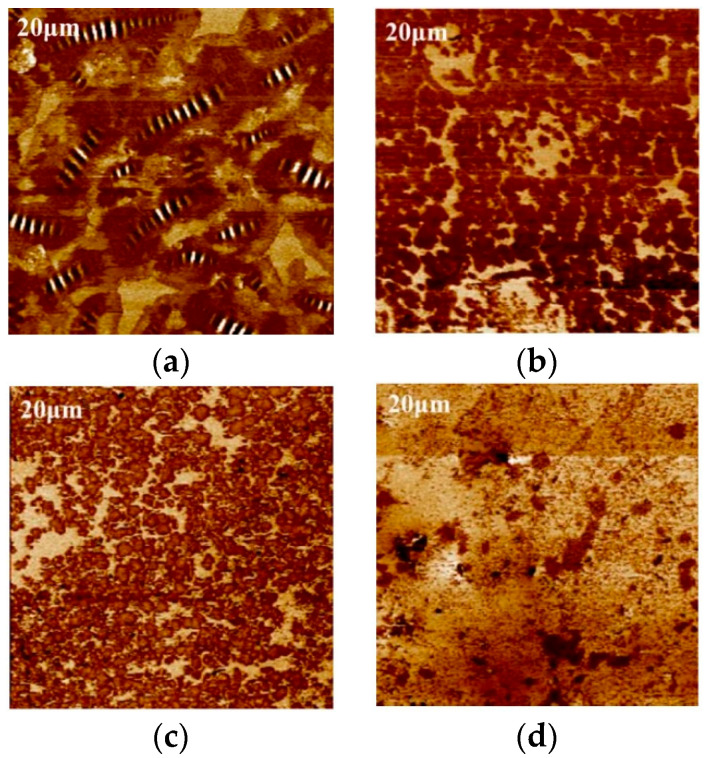
AFM mappings of the asphalt in different states. (**a**) Surface of single asphalt; (**b**) interior of single asphalt; (**c**) interior of asphalt mortar; (**d**) interior of the mixture [29].

It is believed that the bee phase is a unique phase structure on the nanoscale surface of asphalt material. However, as observed in Figure 4, it can be clearly seen that there were a variety of phases in the phase modulus mappings of the asphalt films in the mixture, but no bee phase was found. This was in agreement with previous studies. Xing et al. adopted AFM technology to obtain the nanophase morphologies of the single asphalt sample surface, single asphalt sample interior, asphalt sample interior in mortar, and asphalt sample interior within a mixture, as presented in Figure 5 [29]. The results showed that the bee phase was found only on the surface of a single asphalt material. Therefore, it can be inferred that the nanoscale phase structure inside the asphalt material is different from that of the surface. The reasons for this phenomenon may be related to the origin of the bee phase. Additionally, asphalt within mortar and mixtures will interact with the mineral powder and the mineral aggregate, which will also affect the nanophase morphology and mechanical properties of asphalt.

Finally, it can be seen from the above analysis that qualitative analysis of the phase modulus image is insufficient for establishing a basis to identify the level of asphalt aging. Therefore, it is necessary to discuss the quantitative characterization method of phase modulus based on mathematical statistics.

### 3.2. Phase Modulus Quantization Method

**Figure 6:**
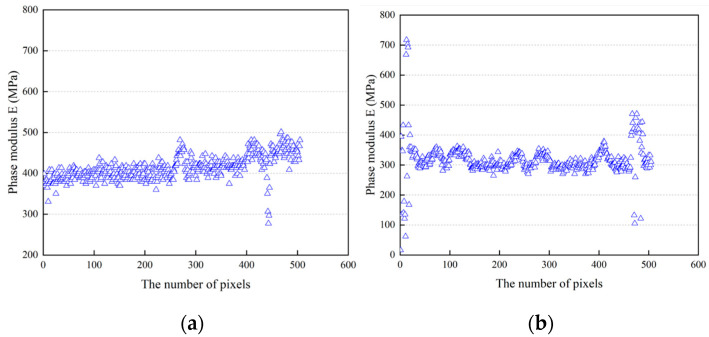

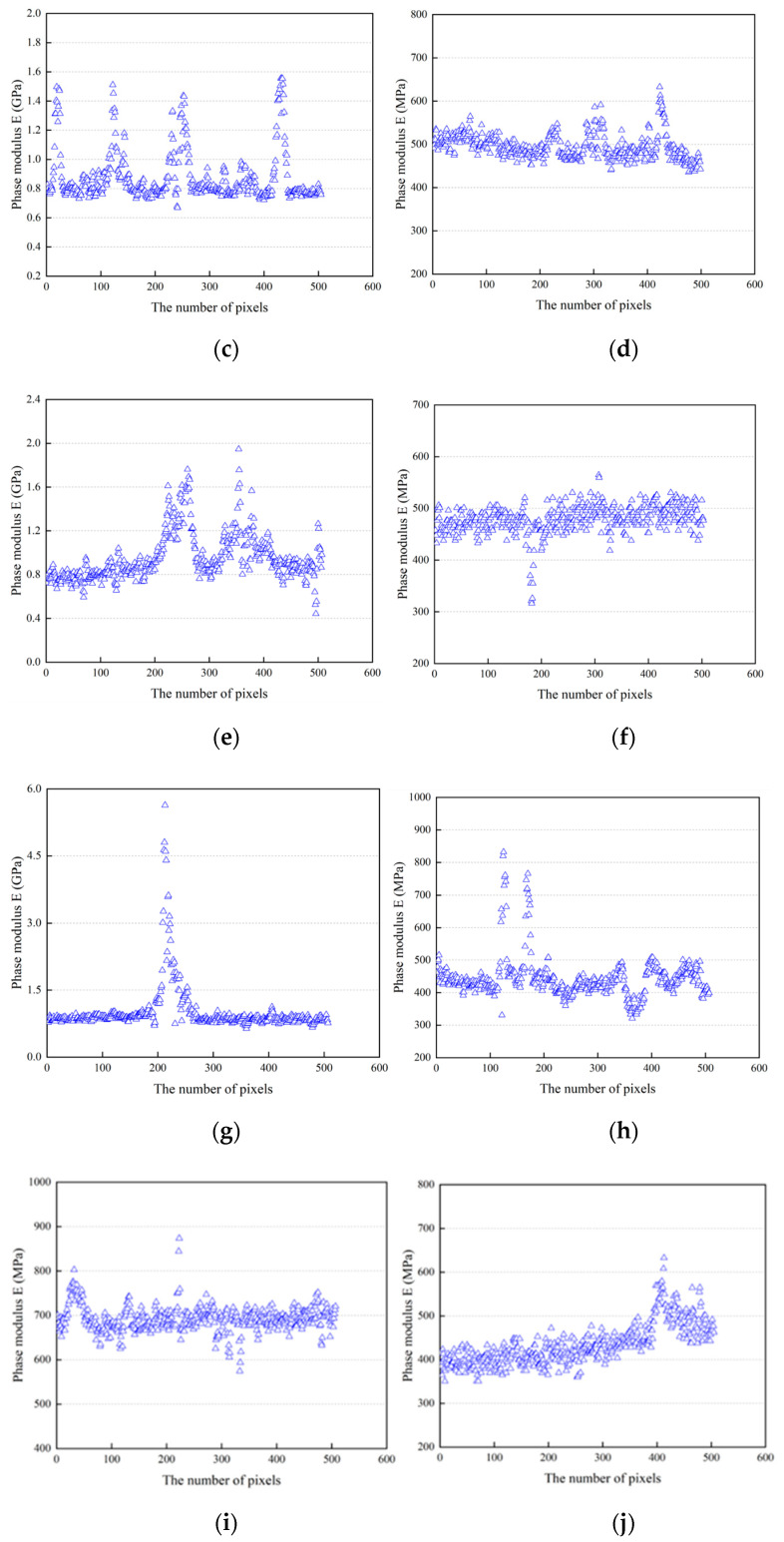
Phase modulus distribution of the asphalt. (**a**) M-1, (**b**) M-2, (**c**) A-1, (**d**) A-2, (**e**) B-1, (**f**) B-2, (**g**) C-1, (**h**) C-2, (**i**) D-1, (**j**) D-2.

In order to quantify the phase modulus of the asphalt within the mixture, the professional nanoscope analysis software (v1.8) was used to extract the DMT modulus values of all pixels in the phase modulus mappings. Then, the distribution diagrams of the phase modulus were plotted, as shown in Figure 6.

**Figure 7:**
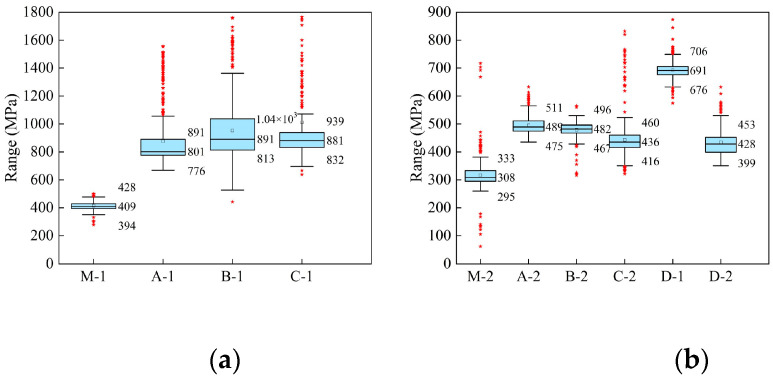
Phase modulus quantization results based on the box plot method. (**a**) SBS-modified asphalt (**b**) base asphalt.

It can be clearly seen that the phase modulus of each sample is distributed across a relatively wide numerical range, and there are also many numerical points with large fluctuations. However, in comparison, the phase modulus values of the laboratory mixture samples were more stable, and those of the on-site pavement coring samples fluctuated greatly. In particular, the phase modulus distribution mappings of the SBS-modified asphalt presented a multi-peak phenomenon. The reason for this phenomenon should be attributed to the influence of mineral material factors and the evolution mechanism of the asphalt phase. It is well known that structural asphalt and free asphalt will be formed in the asphalt–mineral interface area, and their phase modulus will inevitably be different, and even modulus gradients will appear. Moreover, since the AFM-QNM test could accurately identify the mechanical information of different phases, the evolution process of the asphalt phase will inevitably affect the test results. As reported by previous literature [30], the box chart, also known as the box whisker chart, is a statistical tool that can represent the distribution of data, being often used to visually observe the distribution of statistics, as well as comparisons between samples. Therefore, considering the distribution characteristics of the data, the box plot method was introduced in this study. The statistical results are shown in Figure 7.

From Figure 2, the box plot visually displays the central trend and dispersion degree of the numerical distribution of the phase modulus, and the outliers of the phase modulus can also be easily identified. Regarding the box plot method characterization parameters, the height of the box represents the degree of dispersion of the phase modulus data, and the median value represents the centralized tendency of the data. According to this, in terms of the degree of data dispersion, for the SBS-modified asphalt, the box height of the laboratory mixture core sample test results was significantly lower than that of the field core sample. For the base asphalt, the box height of the test results of the laboratory core sample was not significantly different from that of the field core sample. This indicated that the test results of the mixture containing base asphalt were more stable. This also showed once again the complexity of the phase modulus evolution of SBS-modified asphalt. From the point of view of the central trend of the data, the median values of the test results of the four modified bitumen were 409 MPa, 801 MPa, 891 MPa, and 881 MPa, respectively. The median values of the five base asphalts were 308 MPa, 489 MPa, 482 MPa, 436 MPa, 691 MPa, and 428 MPa, respectively. Compared with quantified results from laboratory samples, the phase modulus of SBS-modified asphalt increased by 0.96 times, 1.18 times, and 1.15 times, and that of base asphalt increased by 0.59 times, 0.56 times, 0.42 times, 1.24 times, and 0.39 times, respectively. This indicates that the phase modulus of asphalt film in the pavement core samples was higher than that of the laboratory mixture samples, indicating that the service environment did cause the aging phenomenon of asphalt materials. In addition, comparing the asphalt aging level of the upper layer and the middle layer from the same pavement, it was found that the upper layer was generally higher than that of the middle layer. This finding suggests that there was an aging gradient in the direction of pavement depth. Although the anti-aging ability of SBS-modified asphalt is better than that of matrix asphalt to some extent, the complex service environment of pavement surface still causes the serious aging phenomenon of surface asphalt.

## 4. Macroscopic Properties of Asphalt Recovered from Mixtures

### 4.1. Rheological Properties Test

**Figure 8:**
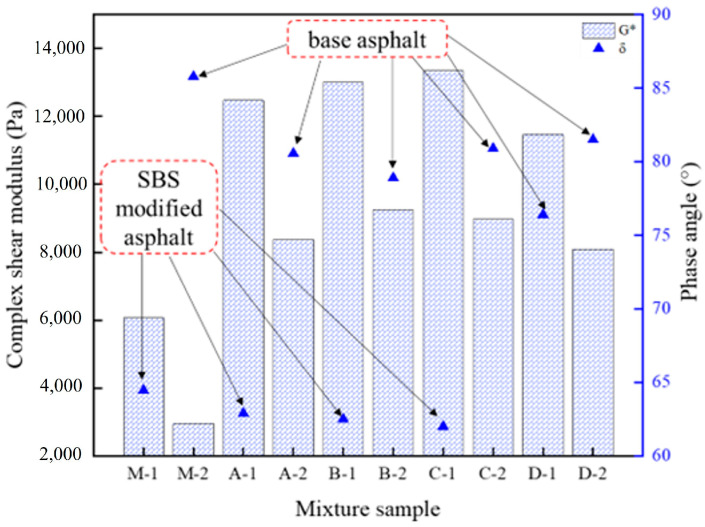
Rheological parameters characterization results of extracted asphalt.

In order to investigate the relationship between the aging level of asphalt in the mixture and the macroscopic properties of a single asphalt material, the asphalt was extracted from the layered cut mixture. Hence, the temperature-scan experiment was carried out at 60 °C. Complex shear modulus and phase angle were selected as characterization parameters of the asphalt’s rheological properties, as presented in Figure 8.

As can be seen from Figure 8, the complex shear modulus of the asphalt in the pavement core samples was significantly larger than that of the laboratory samples. This finding is well correlated with the characterization results of phase modulus, as described above in this paper. Moreover, considering the phase angle, the pavement core samples exhibited lower values compared to the laboratory samples. Specifically, for the laboratory samples, the phase angle of SBS-modified asphalt was 64.47° and that of base asphalt was 85.78°. However, the phase angle values of the SBS-modified asphalt in the pavement core sample were distributed in the range of 62~62.9°, showing obvious polymer rheological properties [22]. This indicates that the elasticity of asphalt in the pavement core sample was highlighted, while the viscosity was reduced, reflecting the performance of asphalt aging.

### 4.2. Penetration Test

In practice, penetration (at 25 °C) degree is usually used as the basis for evaluating the aging level of old asphalt. In this study, the penetration test of extracted asphalt from the mixtures was also tested. The test results, shown in Table 2, clearly indicate that the penetration of asphalt extracted from the pavement core samples significantly reduced compared to that of the laboratory samples.

## 5. The Correlation between Phase Modulus and Macroscopic Properties

**Figure 9:**
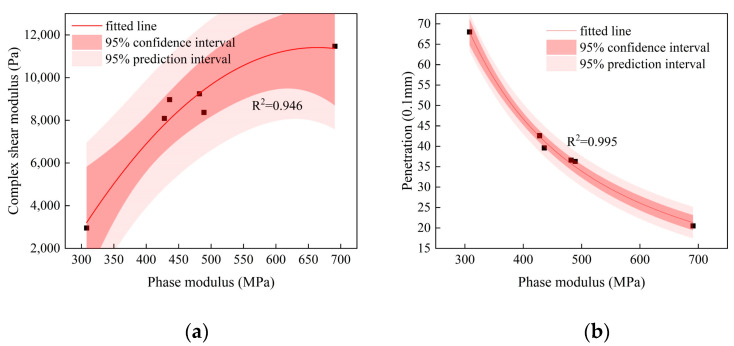
The relationship between phase modulus and performance at macroscale. (**a**) Phase modulus and complex shear modulus. (**b**) Phase modulus and penetration.

It is necessary to discuss the correlation between the phase mechanical indexes of the asphalt film within the mixture and the macroscopic properties of extracted asphalt, in order to provide ideas for the establishment of cross-scale performance models (Equation (2)) of different existing forms of asphalt binders. Considering that the aging behavior of the asphalt phase is affected by asphalt type, the number of the test samples in this study is also limited. Therefore, the mixture samples containing base asphalt were taken as examples. The correlation regression analysis of phase modulus and the complex shear modulus, as well as phase modulus and penetration, were carried out using Origin software (v2022). The results are shown in Figure 9.

(2) $$M=f\left(m\right)$$

where *M* = the macroscopic properties of extracted and recovered asphalt; *m* = the phase properties of the asphalt film in the mixture.

With the increase in phase modulus, the penetration decreased, and the complex shear modulus increased. It can be clearly seen that all points were within the 95% confidence band, indicating that the phase modulus and the complex shear modulus, as well as the phase modulus and penetration, showed a better fitting effect. Moreover, it was noted that the fitting points of the phase modulus and the complex shear modulus were farther from the fitting line, whereas the fitting points of the phase modulus and penetration were closer to the fitting line. This showed a better fit between phase modulus and penetration. However, being limited by the sample quantity, test conditions, and other factors, the universality of this conclusion is still questionable. There is a lot of work to be completed in future studies. It should be noted that this study achieved the goal of identifying the phase mechanical properties of asphalt film in the mixture in situ, and attempted to establish its relationship with conventional indexes. It also provided innovative ideas for the future research on multi-scale numerical simulation or the establishment of cross-scale performance models. In addition, this study also suggests that advanced characterization techniques, such as nano-infrared should be applied to the testing and characterization of asphalt film properties in the mixture, and the test results should be combined with those from this study, which may provide an innovative way to identify the degree of asphalt aging in the old pavement mixture.

## 6. Conclusions

The objective of this study was to recognize and characterize the nanoscale phase modulus of asphalt film within pavement mixture cores using AFM-QNM technology. The core samples from the upper layers and middle layers of four highways and laboratory samples were taken as the research object. The correlation between the phase modulus and the macroscale properties of recovered asphalt from the mixture cores were investigated with the help of the method of mathematical statistics. The following conclusions can be drawn:

(1) Compared with the laboratory samples (namely, M), the characteristics of the multi-phase and diversified phase of the pavement core samples (namely, A, B, C, and D) were more significant. This phenomenon indicates that the asphalt in the pavement core samples had an obvious phase separation phenomenon due to aging. Further, the phase similar to black spots was found in the phase modulus mappings of the SBS-modified asphalt, but this phenomenon was not obvious for base asphalt.
(2) The phase modulus of each sample was distributed across a relatively wide numerical range, and there were also many numerical points with large fluctuations. Especially for the mixture sample containing SBS-modified asphalt, the phase modulus distribution mappings presented a multi-peak phenomenon. Hence, considering the distribution characteristics of the data, the box plot method was introduced in this study.
(3) Compared with the quantified results from the laboratory samples, the phase modulus of SBS-modified asphalt increased by 0.96 times, 1.18 times, and 1.15 times, and that of base asphalt increased by 0.59 times, 0.56 times, 0.42 times, 1.24 times, and 0.39 times, respectively. This showed that the aging degree of asphalt in the upper layer was generally greater than that in the middle layer. This finding also suggests that there was an aging gradient in the direction of pavement depth.
(4) It can be clearly seen that all points were within the 95% confidence band, indicating that the phase modulus and the complex shear modulus, as well as the phase modulus and penetration, showed a better fitting effect. Moreover, it was noted that the fitting points of the phase modulus and the complex shear modulus were farther from the fitting line, whereas the fitting points of the phase modulus and penetration were closer to the fitting line. This showed a better fit between the phase modulus and penetration.
(5) However, limited by the sample quantity and test conditions, as well as by other factors, the universality of this conclusion is still questionable. It should be noted that this study achieved the goal of identifying the phase mechanical properties of asphalt film in the mixture in situ. In addition, this study also suggests that advanced characterization techniques, such as nano-infrared, should be applied to the testing and characterization of asphalt film properties in the mixture, and the test results should be combined with those from this study. This may provide an innovative way to identify the degree of asphalt aging in older pavement mixtures.

## Figures and Tables

**Table 1:** Information on the pavement structure.

Pavement	Service Life (Years)	Asphalt Type/Designation	
		Upper Layer	Middle Layer
M	0	SBS-modified asphalt/M-1	Base asphalt//M-2
A	6	SBS-modified asphalt/A-1	Base asphalt /A-2
B	6	SBS-modified asphalt/B-1	Base asphalt//B-2
C	7	SBS-modified asphalt/C-1	Base asphalt//C-2
D	4	Base asphalt/D-1	Base asphalt//D-2

**Table 2:** The test results of penetration (25 °C, 0.1 mm).

Mixture Sample	M	A	B	C	D
Upper layer	56.0	32.5	30.4	28.1	20.5
Middle layer	68.0	36.3	36.6	39.6	42.6

## Data Availability

The raw data supporting the conclusions of this article will be made available by the authors on request.
